# Long-term application of organic manures and chemical fertilizers improve the organic carbon and microbiological properties of soil under pearl millet-wheat cropping system in North-Western India

**DOI:** 10.1016/j.heliyon.2024.e25333

**Published:** 2024-01-30

**Authors:** Manju Kumari, Sunita Sheoran, Dhram Prakash, Dharam Bir Yadav, Parmod Kumar Yadav, Mukesh Kumar Jat, Ankit, Apurva

**Affiliations:** a Indian Agricultural Research Institute, New Delhi, 110012, India; b Chaudhary Charan Singh Haryana Agricultural University, Hisar, Haryana, 125004, India; c Central Soil Salinity Research Institute, Karnal, Haryana, 132001, India

**Keywords:** Long term, Organic manure, Fertilizer, Organic carbon fractions, Enzyme activities, Pearl millet-wheat

## Abstract

An on-going long term field experiment started in *Rabi* 1995 at the Research Farm of the Department of Soil Science, Chaudhary Charan Singh Haryana Agricultural University, Hisar, Haryana (India) under the pearl millet-wheat cropping system was selected to study the effect of long-term application of organic manures and fertilizers on soil organic carbon and microbiological properties. Highest soil organic carbon (SOC: 1.18 %), dissolved OC (DOC: 64.74 mg kg^−1^) content, microbial biomass C (MBC: 618.40 mg kg^−1^), dehydrogenase (DHA: 72.83 μg TPF g^−1^ 24 hr^−1^), alkaline phosphatase (APA: 685.44 μg PNP g^−1^ soil hr^−1^) and aryl sulfatase (ASA: 12.56 μg PNP g^−1^ soil hr^−1^) activities were observed with the application of 15 Mg FYM+150 kg N+30 kg P_2_O_5_ ha^−1^. Integrated application of chemical fertilizers with pressmud showed superiority in the improvement of microbial biomass nitrogen (MBN: 73.73 mg kg^−1^) and urease activity (69.54 μg NH_4_^+^ g^−1^ hr^−1^) than FYM or poultry manure plus NP. Beneficial impacts of the sole application of organic manures on SOC, DOC, MBC content, DHA, APA, and ASA were found in order as: FYM > pressmud > poultry manure. Impacts of nutrient management practices on soil carbon fractions decreased with depth. Poultry manure application, either alone or in combination with NP fertilizers was inferior to FYM and pressmud. The SOC had a positive relationship with MBC (R^2^ = 0.95) and MBN (R^2^ = 0.75) and, also showed a highly positive and significant correlation with microbiological properties of soil. This dynamic equilibrium among soil properties indicated that the nutrient management practices that improve SOC could lead to improve soil fertility and accrued microbiological properties in these soils. This study revealed that conjuctive use of organic manures and chemical fertilizers have positive impact on soil fertility and microbiological properties as compared to sole application of organic manures or fertilizers; and among organic manures, FYM was superior to pressmud followed by poultry manure.

## Highlights

• Long-term use of manures with fertilizers improved the soil in the pearl millet-wheat cropping system.
• The impact of manures on soil was in the order of FYM > pressmud > poultry manure.
• Pressmud was better than other manures for MBN and Urease activity.
• Soil OC had a strong positive relationship with microbial N & C.
• Nutrition practice improving SOC, gave better soil microbial & fertility status.

## Graphical abstract

**Graphical abstract**: The subscript figures in treatment indicate the dose of nitrogen (N) and phosphorus (P_2_O_5_) in kg ha^−1^, farmyard manure (FYM), poulty manure (POM) and Pressmud (PRM) in Mg ha^−1^.

**Figure:**
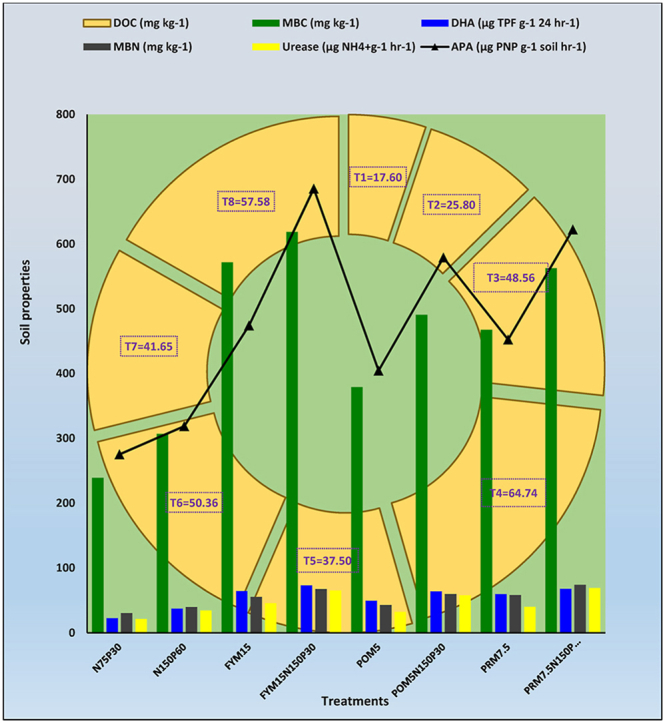

## 1 Introduction

Soil organic carbon (SOC) influences dynamics of nutrient availability and microbiological activities of soil, and plays germane role in maintaining soil fertility. In semi-arid areas of India, soil organic matter (SOM) quickly decomposes and exacerbates its depletion [1,2]. Imbalanced nutrient application facilitates the deterioration in soil properties [3]. Application of organic manures improves soil properties *i.e.* nutrient holding and buffering capacity, which in turn enhances the microbial activities [4]. Organic manures like farm yard manure (FYM), pressmud and poultry manure are available nutrient sources and recognized to enhance the SOC and accessible nutrient contents over fertilizers application [[5], [6], [7], [8], [9]]. No doubt, inorganic fertilizers provide nutrients in readily available form but their excessive use could have inhibitory effects on microbial as well as enzymatic activities. However, combined supply of organic manures along with chemical fertilizers has been suggested to maintain soil fertility and microbial activity [10]. Adoption of balanced use of chemical fertilizers coupled with FYM improves physical condition of soils and enhanced the crop productivity [11].

Dissolved organic carbon (DOC) represents a fraction of labile C, an array of simple organic molecules in dissolved form of SOC, and remains in equilibrium with solid phase of soil [12]. The DOC has rapid turnover rate and has been suggested as a sensitive indicator of management induced changes in SOM. It plays an important role in soil nutrients mobility and their availability to plant. The DOC has also been considered a labile substrate for microbial activity [10,13,14]. Dehydrogenase enzyme lies in viable cells and represents total span of oxidative activity of soil micro flora which helps in transformation of different plant nutrients in soil [[15], [16], [17], [18]]. Urease existing as extracellular enzyme, mainly originates from plant and microorganisms. The activity of urease *i.e.* hydrolysis of urea is increased by addition of organic manures. The organic sulphur gets mineralized to SO_4_^2−^ through hydrolysis of organic sulfate esters by aryl-sulfatase enzyme and play pertinent role in sulphur cycling in soils [19]. Long-term use of chemical fertilizers and organic manures are important tool to determine the dynamics of soil characteristics.

Soil OC content has been found to increase with continuous application of organic manure on long-term basis in China [20]. It is also found to be increased with increasing doses of fertilizers and conjuctive use of FYM along with recommended dose of fertilizers (RDF) in long-term field experiments under subtropical climate [21]. Long-term effect of manures and fertilizers on DOC content of soil has been well documented in the rice-potato-wheat and the maize-potato-onion cropping systems in India with the highest DOC content under 100 % chemical N + vermicompost + crop residues [22]. Positive impact of integrated nutrient management (INM) on water soluble carbon has also been observed in the rice-wheat-jute cropping system [12]. Long-term (32 years) integration of 50 % RDF + 2.5 t ha^−1^ FYM and 50 % RDF + 0.5 t ha^−1^ azolla had significant positive impact on SOC and microbial biomass carbon (MBC) in acid soil of North- Eastern India under rice-rice cropping system [23]. Similarly, application of vermicompost + poultry manure also gave highest MBC, microbial biomass nitrogen (MBN) and dehydrogenase activity in cold region of Jammu, India [24].

Microbial biomass carbon (MBC) has been found as more sensitive indicator than soil organic carbon content of soil [25] and organic amendments have significant effect on MBC and MBN contents [26,27]. Substitution of mineral fertilizers with a higher ratio (80 %) of organic manures resulted in higher MBC and MBN than a lower ratio (20–60 %) of substitution [28].

Continuous application of FYM has been found to increase the dehydrogenase activity at various growth stages of rice under rice-rice cropping systems in semi-arid region of South India [29]. Integrated nutrient management (INM) with the inclusion of organic manures like poultry manure, urban compost, maize residue, green manures, FYM, and press mud also resulted in higher alkaline phosphatase, arylsulfatase, and dehydrogenase activities under different cropping systems [5,[30], [31], [32], [33]]. Plenty of information exists about the impacts of FYM and its conjoint application with chemical fertilizers on soil fertility for temperate to tropical regions [[34], [35], [36], [37], [38], [39]].

It is hypothesized that a field would show accrual in soil microbiological properties which would directly or indirectly lead to improve soil fertility when the addition of manures in conjunction with chemical fertilizers would be done. Short-term experiments would not give confident results regarding pros and cons of the application of organic manures alone or combined with chemical fertilizers on soil microbial properties. However, sufficient information on the comparative impacts of organics (FYM, poultry manure and pressmud) and fertilizers application in pearl-millet-wheat cropping systems long-term basis under semi-arid conditions similar to north-western India is lacking. Such information needs to be generated from long-term experimentation under these situations for precise quantification.

Considering the above facts, studies on soil properties were undertaken from an already running long-term experiment (25 years) at Hisar, India, with the main aim to measure the impact of long-term application of organic manures and fertilizers on SOC, microbial biomass C, N and enzyme activities in pearl millet-wheat cropping system under semi-arid eco-regions of North-West India.

## 2 Materials and methods

### 2.1 Study location, treatment details, and design

A long-term field experiment under pearl-millet-wheat cropping system was started in the winter season 1995 at the Research Farm of the Department of Soil Science, CCS Haryana Agricultural University, Hisar, Haryana. The experimental site is located at 29^o^16′ N and 75^o^7′ E, in a semi-arid zone of India with average annual rainfall and temperature of 443 mm and 24.8 °C, respectively. The soil was coarse loamy (*Typic Ustochrept*) in texture with 0.39 % initial soil organic carbon (SOC). The present investigation was undertaken during the 25th cycle of pearl -millet-wheat cropping system in 2020. The present research experiment comprised total of eight treatments involving application of nitrogen (N) through urea and phosphorus through fertilizer P along with organic manures. The treatment combinations were: T_1_: N_75_P_30_ (75 kg N+30 kg P_2_O_5_ ha^−1^), T_2_: N_150_P_60_ (150 kg N+60 kg P_2_O_5_ ha^−1^), T_3_: FYM_15_ (15 Mg FYM ha^−1^), T_4_: FYM_15_N_150_P_30_ (15 Mg FYM+150 kg N+30 kg P_2_O_5_ ha^−1^), T_5_: POM_5_ (5 Mg Poultry manure ha^−1^), T_6_: POM_5_N_150_P_30_ (5 Mg Poultry manure+150 kg N+30 kg P_2_O_5_ ha^−1^), T_7_: PRM_7.5_ (7.5 Mg pressmud ha^−1^), T_8_: PRM_7.5_N_150_P_30_ (7.5 Mg Pressmud+150 kg N+30 kg P_2_O_5_ ha^−1^). These treatments were replicated three times on the permanent plots in a randomized block design (RBD). The FYM, POM, and PRM were applied once in a year at the time of wheat sowing. The same dose of chemical fertilizers was applied in both crops (pearl millet and wheat) as per the treatments. The FYM, POM and PRM had organic carbon 38.70, 37.10 and 39.60 %, nitrogen 0.74, 0.82 and 0.95 %, phosphorus 0.45, 1.26 and 1.10 %, and potassium 1.58, 1.37 and 0.96 %, respectively (Table 1).

**Table 1:** Nutrient composition of various organic manures used.

Organic sources	Organic carbon (%)	Nitrogen (%)	Phosphorus (%)	Potassium (%)
Farmyard manure	38.70	0.74	0.45	1.58
Poultry manure	37.10	0.82	1.26	1.37
Pressmud	39.60	0.95	1.10	0.96

### 2.2 Soil sampling and analysis

Two sets of soil samples were drawn from each plot after the harvesting of wheat (April 2020). Sampling was done from surface (0–15 cm) and subsurface (15–30 cm) in triplicate from each treatment. One set of soil samples was air-dried, ground passed through a 2 mm sieve and stored in a plastic bag in ambient conditions for chemical analysis later on. Another set of undried fresh soil samples was stored in a 4 °C freezer for analysis of biological properties. The microbial properties (microbial biomass carbon: MBC, microbial biomass nitrogen: MBN, dehydrogenase activity: DHA, alkaline phosphatase activity: APA, aryl sulfatase activity: ASA and urease activity) were analyzed for surface soil (0–15 cm) only and their results were expressed on the oven dry weight basis. Other parameters (SOC and Dissolved OC) were analyzed for 0–15 and 15–30 cm layers. The SOC content of soil was determined by using the wet digestion method [40]. The DOC content was estimated by using the dichromate acid oxidation method [41].

Among soil microbial properties, the soil MBC content was estimated by the chloroform fumigation method [42] and MBN content was measured by chloroform fumigation followed by the Kjeldahl distillation method [43]. Soil dehydrogenase was assayed by estimating the rate of production of tri-phenyl formazan (TPF) from 2,3,5-triphenyl tetrazolium chloride (TTC) [44]. Alkaline phosphatase activity was tested by incubating the soil with a *p*-nitrophenyl phosphate (PNP) solution and measuring the intensity of colour developed [45]. Urease activity was determined by extracting the soil with 2 M potassium chloride-phenyl mercuric acetate (KCl-PMA) solution followed by colour development [46]. The activity of aryl sulfatase enzyme was measured by estimating the p-nitro-phenol released when the soil was incubated with potassium *p*-nitrophenyl sulfate solution [47].

### 2.3 Statistical analysis

Statistical analyses for comparing means and correlation among different parameters were accomplished using the program STATISTICA 6.0 Stat Soft, Inc. (2001). The means were compared using the least significant difference (LSD) test at P = 0.05. Mean separation for different treatments was also evaluated using Duncan's multiple range test (DMRT) with SPSS 16.0 for Windows (SPSS Inc., Chicago, U.S.A.).

## 3 Results and discussion

### 3.1 Soil organic and dissolved organic carbon content in the soil

After 25 years of continuous pearl-millet-wheat cropping system, the SOC content slightly decreased under treatment T_1_ (N_75_P_30_) (0.37 %) as compared to initial value (0.39 %). The highest SOC in 0–15 cm (1.18 %) and in 15–30 cm (0.59 %) soil depth was recorded with the addition of FYM_15_N_150_P_30_ (Table 2). Similarly, the DOC was also highest under FYM_15_N_150_P_30_ *i.e*. 64.74 mg kg^−1^ in 0–15 cm soil depth and 39.65 mg kg^−1^ in 15–30 cm soil depth. It indicated the decreasing trend of SOC and DOC with soil depth. Comparatively less content of SOC and DOC in subsurface soil than surface soil might be attributed to the continuous addition of organic matter in plough layer via different organic manures year after year and root deposition by crops. The addition of FYM liberates more DOC in soil, and the compact layer underneath the soil does not allow its leaching hence DOC remained higher in surface soil [48]. The treatment receiving N_150_P_60_ (T_2_) showed more SOC and DOC content than N_75_P_30_ (T_1_). The higher root biomass and crop residue or stubble retention in better-fertilized plots might have increased SOC content [49,50].

**Table 2:** Effect of long-term manure and fertilizer application on soil organic carbon (SOC) and dissolved organic carbon (DOC) content in surface and sub-surface soil in pearl millet-wheat cropping system.

Treatment						SOC (%)		DOC (mg kg^−1^)	
Reference/acronym		Manure		Fertilizer					
		Type of manure	Dose (Mg ha^−1^ yr^−1^)	N (kg ha^−1^)	P_2_O_5_ (kg ha^−1^)	0–15 cm	15–30 cm	0–15 cm	15–30 cm
T_1_	N_75_P_30_	No manure	0	75	30	0.37^a^	0.23^a^	17.60^a^	11.60^a^
T_2_	N_150_P_60_		0	150	60	0.46^a^	0.27 ^b^	25.80 ^b^	15.46 ^b^
T_3_	FYM_15_	Farmyard manure	15	0	0	1.06^e^	0.52^f^	48.56^e^	31.80^e^
T_4_	FYM_15_N_150_P_30_		15	150	30	1.18^f^	0.59 ^g^	64.74 ^g^	39.65^f^
T_5_	POM_5_	Poultry manure	5	0	0	0.75 ^b^	0.32^c^	37.50^c^	18.12^c^
T_6_	POM _5_N_150_P_30_		5	150	30	0.81 ^bc^	0.37 ^d^	50.36^e^	22.88 ^d^
T_7_	PRM_7.5_	Pressmud	7.5	0	0	0.89 ^cd^	0.40 ^d^	41.65 ^d^	21.53 ^d^
T_8_	PRM_7.5_N_150_P_30_		7.5	150	30	0.95 ^de^	0.46^e^	57.58^f^	31.46^e^
	LSD (P = 0.05)					0.13	0.04	4.08	2.35

Abbreviations: N: Nitrogen, P: Phosphorus, FYM: Farmyard manure, POM: Poultry manure, PRM: Pressmud. Mean values within a column followed by different letters differ significantly (*p < 0.05*) by Duncan's multiple range test (DMRT).

The higher SOC and DOC content with the addition of organic manure along with NP fertilizers could be the result of the priming effect of applied N. Nitrogen application stimulates the microbial activities which could have led to a commensurate increase in DOC content during the decaying of organic matter [51]. The higher crop productivity and enhanced rhizospheric activities could be responsible for the accretion of OC fractions in treatments receiving organic manures and mineral fertilizers [12]. Regardless of the soil depth, the application of FYM resulted in higher SOC and DOC content followed by PRM and POM. This differential effect could be due to the variation in SOM added through manures, their nutrient constitution and rate of decomposition in soil [52,53]. Integrated use of manures along with NP fertilizers showed superiority for improving SOC and DOC. Manures add more SOC in soil which could be attributed to the presence of more humified, less labile and labile forms of C in decomposed organic materials as supported by findings of earlier workers [10,12,49,52,54].

### 3.2 Microbial biomass carbon (MBC) and microbial biomass nitrogen (MBN) in soil

**Fig. 1:**
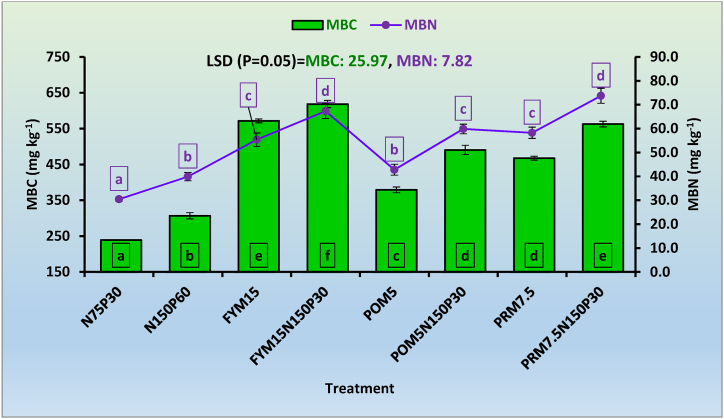
Impact of long-term manure and fertilizer application on microbial biomass carbon (MBC) and microbial biomass nitrogen (MBN) content. The subscript figures in treatment indicate the dose of nitrogen (N) and phosphorus (P_2_O_5_) in kg ha^−1^, FYM, POM, and PRM in Mg ha^−1^. Mean values for a soil property followed by different letters differ significantly (*p < 0.05*) by Duncan's multiple range test (DMRT)].

**Fig. 2:**
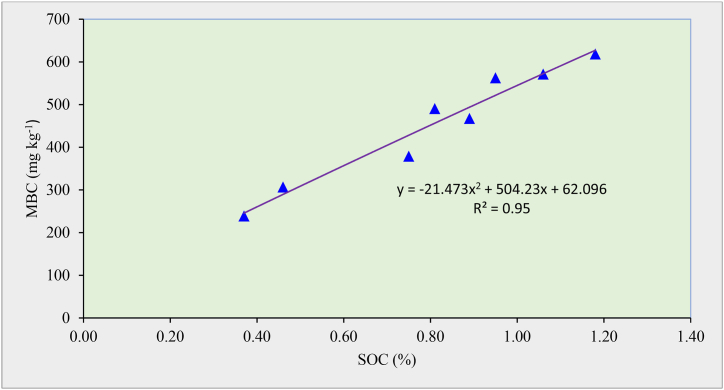
Relationship between soil organic carbon (SOC) and microbial biomass carbon (MBC) content of soil.

The post-harvest MBC content in soil ranged between 238.93 and 618.40 mg kg^−1^ among various treatments (Fig. 1). The highest (618.40 mg kg^−1^) MBC content was observed with the addition of FYM along with chemical fertilizers (T_4_) and lowest (238.93 mg kg^−1^) under half dose of NP fertilizers alone (T_1_). A significantly higher MBC content was recorded under the incorporation of organic manures over chemical fertilizer treatments. Sole application of manures *i.e.* FYM_15_, POM_5_ and PRM_7.5_ showed 86.3, 23.5, and 52.3 % higher MBC content over the treatments receiving recommended dose of NP fertilizers, respectively. Application of N fertilizers (urea) may produce an acidifying environment which might have adverse effects on different types of microorganisms and thus result in lower MBC content in chemical fertilizer treatments [55]. The FYM receiving treatments recorded the higher MBC content as compared to PRM and POM. This could be due to the large amount of SOM added through FYM as compared to POM or PRM [56]. Higher value of labile C/DOC in soil dressed with FYM led to greater plant growth, root biomass, enhanced root exudation, increased microbial activity. It appeared as an immediate substrate for soil micro-organisms and thus showed higher MBC [12]. In this study, a polynomial relationship between SOC and MBC content of soil was also observed having R^2^ = 0.95 (Fig. 2).

**Fig. 3:**
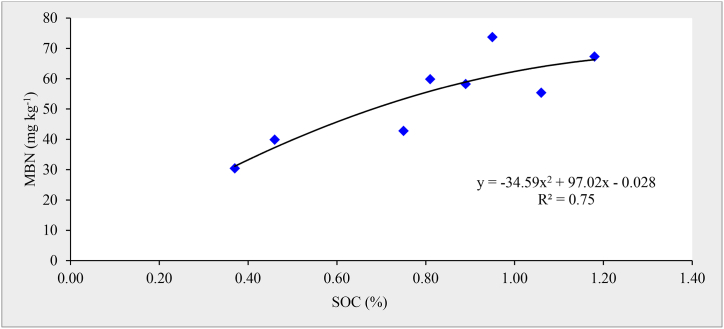
Relationship between soil organic carbon (SOC) and microbial biomass nitrogen (MBN) content of soil.

The soil MBN content ranged between 30.44 and 73.73 mg kg^−1^ among various treatments (Fig. 1). The highest (73.73 mg kg^−1^) MBN content in soil was recorded with the addition of 7.5 Mg PRM+150 kg N+30 kg P_2_O_5_ ha^−1^ (T_8_) and lowest (30.44 mg kg^−1^) under N_75_P_30_ (T_1_). The treatments receiving organic manures (FYM and PRM) showed significantly higher MBN content in soil in comparison to chemical fertilizers applied alone. Application of FYM, POM, and PRM led to 38.8, 7.2 and 46.0 % higher MBN content over the recommended dose of fertilizer (T_2_) and, 81.9, 40.5 and 91.4 % higher MBN over 50 % RDF (T_1_), respectively. The higher values of MBC and MBN in organically amended treatments could be considered beneficial for microbial proliferation and favourable for their enhanced activities [57]. The conjunctive application of manures and fertilizers recorded significantly higher MBN content than sole application of organic manures. It could be due to the addition of higher SOC content and more root expansion coupled with better plant growth under the integrated application of NP through chemical sources [55]. Among the organically dressed treatments, the highest MBN content was observed with the addition of PRM as compared to FYM or POM. It could be due to the fact that the PRM is a by-product of sugar factories and contains more easily decomposable sugar compounds which enhanced the microbial activities. Also, PRM contains more lignocellulosic compounds which are vital sources of carbon for the microbial decomposition of SOM [58]. The integrated nutrient applications showed favourable impacts on MBN in soil. A polynomial relationship between SOC and MBN content of soil was observed having R^2^ = 0.75 (Fig. 3).

### 3.3 Soil enzymatic activities

The values of dehydrogenase enzyme activity (DHA) varied between 22.54 and 72.83 μg TPF g^−1^ 24 hr^−1^ among various treatments (Table 3). The highest (72.83 μg TPF g^-1^24 hr^−1^) dehydrogenase activity was recorded with the application of FYM in combination with NP fertilizers treatment (T_4_). Treatments receiving FYM_15,_ POM_5_ and PRM_7.5_ alone exhibited 73.3, 33.2, and 61.0 % higher dehydrogenase activity over treatment with recommended dose of NP fertilizers (T_2_), respectively. The addition of FYM_15_N_150_P_30_, POM_5_N_150_P_30,_ and PRM_7.5_N_150_P_30_ recorded 95.7, 71.4 and 81.2 % higher dehydrogenase activity over the recommended dose of NP fertilizers, respectively. Thus, the application of organic manures either alone or in conjunction with chemical fertilizers led to significantly higher dehydrogenase enzyme activity over soils dressed with chemical fertilizers only. Higher values of DHA could be due to decomposition of applied organic material by the intra and extracellular enzymes and increased microbial activity in the soil [59]. Rapidly decaying organic materials could have a strong effect on the metabolism of living organisms and the dehydrogenase activity of soil [60]. More easily mineralizable components of different nutrients in bulk soil solution quicken the microbial growth and their multiplicity which accompanies higher DHA [34]. The lowest enzyme activity under NP fertilizer treatments might be due to less amount of SOM and DOC present in these treatments. Addition of FYM_15_N_150_P_30_, POM_5_N_150_P_30_ and PRM_7.5_N_150_P_30_ recorded 12.9, 28.6 and 12.5 % higher DHA over FYM_15_, POM_5_ and PRM_7.5_, respectively. It could be attributed to additional contribution of nutrients to soil in form of N and P through fertilizers which could narrow down the C: N ratio and enhance enzymatic activity in soil. The application of mineral fertilizers in conjunction with organics enriched the DHA in soils due to abundance of labile nutrients which were restored by living microorganisms [34,61].

**Table 3:** Impact of long-term manure and fertilizer application on soil enzymatic activities (0–15 cm).

Treatment						Enzymatic activities			
Reference/acronym		Manure		Fertilizer		DHA (μg TPF g^-1^24 hr^−1^)	Urease (μg NH_4_^+^g^−1^ hr^−1^)	APA (μg PNP g^−1^ soil hr^−1^)	ASA (μg PNP g^−1^ soil hr^−1^)
		Type of manures	Dose (Mg ha^−1^ yr^−1^)	N (kg ha^−1^)	P_2_O_5_ (kg ha^−1^)				
T_1_	N_75_P_30_	No manure	0	75	30	22.54^a^	21.45^a^	275.20^a^	3.08^a^
T_2_	N_150_P_60_		0	150	60	37.21 ^b^	34.32 ^b^	318.75 ^b^	5.29 ^b^
T_3_	FYM_15_	Farmyard manure	15	0	0	64.50 ^d^	45.66 ^d^	474.12 ^d^	7.96^c^
T_4_	FYM_15_N_150_P_30_		15	150	30	72.83^e^	66.13^f^	685.44 ^g^	12.56^e^
T_5_	POM_5_	Poultry manure	5	0	0	49.58^c^	32.44 ^b^	404.45^c^	7.05^c^
T_6_	POM_5_N_150_P_30_		5	150	30	63.78 ^d^	58.26^e^	578.96^e^	11.26 ^d^
T_7_	PRM_7.5_	Pressmud	7.5	0	0	59.91 ^d^	41.33^c^	452.38 ^d^	7.35^c^
T_8_	PRM_7.5_N_150_P_30_		7.5	150	30	67.42 ^de^	69.54^f^	622.36^f^	10.21 ^d^
	LSD (P = 0.05)					8.18	5.69	28.35	1.21

Abbreviations: N: Nitrogen, P: Phosphorus, FYM: Farmyard manure, POM: Poultry manure, PRM: Pressmud, DHA: Dehydrogenase activity, APA: Alkaline phosphatase activity, ASA: Aryl sulfatase activity, TPF: Triphenylformazan, PNP: Para nitrophenol. Mean values within a column followed by different letters differ significantly (*p < 0.05*) by Duncan's multiple range test (DMRT).

The highest and lowest urease activity was recorded with the application of PRM_7.5_N_150_P_30_ (69.54 μg NH_4_^+^ g^−1^ hr^−1^) and N_75_P_30_ (21.45 μg NH_4_^+^ g^−1^ hr^−1^) treatments, respectively. Significantly higher urease activity in soil was observed with the addition of N_150_P_60_ (34.32 μg NH4^+^ g^−1^ hr^−1^) in comparison to N_75_P_30_ treatment (21.45 μg NH4^+^ g^−1^ hr^−1^). Evidently, this might be owing to the supply of more N through nitrogenous fertilizer (urea). Among various treatments, the plots receiving PRM coupled with NP fertilizers showed the highest (69.54 μg NH4^+^ g^−1^ hr^−1^) urease activity because of more N percentage in PRM (Table 1). Pressmud is a by-product of sugarcane and is rich in nitrogen due to sugarcane being a high N feeding crop, which could lead to enhanced urease activity in PRM-treated plots [56]. The greater urease activity might be expected due to accrued SOC content (Table 2) in soil that increased microbial population and MBC (Fig. 1) thereby stimulating the secretion of urease enzyme. Except sole application of POM (T_5_), all the treatments significantly increased the urease activity over half the dose of NP (T_1_). Application of manures coupled with NP fertilizers showed significant accrual in urease activity as compared to sole application of manures, thus, indicating the favourable impact of combined application of manures and NP fertilizers on urease activity.

A significant increase in the alkaline phosphatase activity (APA) was exhibited in all other treatments as compared to half the dose of RDF (T_1_) (Table 3). The highest APA activity (685.44 μg PNP g^−1^ soil hr^−1^) was recorded with the addition of FYM in combination with chemical fertilizers (T_4_). Higher APA activity was recorded with the application of FYM followed by PRM and POM. This increase in APA may be due to greater accrual of SOC in treatments receiving FYM either alone or in combination with mineral nutrients *i.e*. NP fertilizers (Table 2) and higher value of available P (Data not given). Conjunctive use of manures and NP fertilizers showed significantly higher APA activity over the sole application of organic manures or NP fertilizers. Decomposition of SOM in the presence of inorganic N and P tends to release certain organic acids which stimulates mineralization and liberation of fixed P to the equilibrium soil solution. An increase in the available P in soil could lead to enhanced alkaline phosphatase activity in soil [62,63]. The adoption of integrated use of manures and mineral fertilizers produces easily decomposable substrates abundantly and encourages higher hydrolysis of esters and hydrides of phosphoric acid which in turn leads to beneficial impact on APA [5]. Improvement in APA under combined application of chemical fertilizers and FYM was ascribed to sustainable maintenance of SOC above antecedent level and improved microbial vividness and growth [64,65].

The highest aryl sulfatase activity (ASA: 12.56 μg PNP g^−1^ soil hr^−1^) was obtained with the addition of FYM in conjunction with NP fertilizers (Table 3). It could be due to the production of various organic acids during FYM decomposition which reduces the soil pH and promotes aryl sulfatase activity in soil. A significantly higher ASA in soil was observed in N_150_P_60_ treatment (5.29 μg PNP g^−1^ soil hr^−1^) over N_75_P_30_ fertilizers (3.08 μg PNP g^−1^ soil hr^−1^). Therefore, indicating the favourable impacts of recommended and balanced fertilizer application on ASA. Application of organic manures alone or in conjunction with NP fertilizers showed a significantly higher ASA than sole application of NP fertilizers. It might be attributed to higher accretion of SOC in organically managed plots in the presence of chemical fertilizers. The SOC acts as heat insulator and protects the extracellular aryl sulfatase from denaturation by high soil temperatures and large diurnal fluctuations in semi-arid tropics [66]. The lowest ASA (3.08 μg PNP g^−1^ soil hr^−1^) was observed with the application of half dose of RDF (T_1_) application. This lower value might be due to less substrate availability for microorganisms in these plots.

### 3.4 Correlation

There was a positive and highly significant correlation among all the soil properties (Table 4). The SOC content was positively and significantly correlated (r > 0.94) with DOC, MBC, and dehydrogenase activity. However, it was least correlated (r = 0.78) with urease as compared to other soil properties. It indicated how SOC impacts N availability under the semi-arid eco-region. The positive and highly significant correlation of SOC and MBC content of soil could be due to improved soil health and favourable environment for microbes growth. Soil enzymes are secreted through microorganisms, dead animals and plants present in the soil. Organic manures enhance the carbon sources for microorganisms thereby making available the energy for their metabolic and reproduction process. It enhances the enzymatic activities in soil. The quick decomposition of SOM provides more substrate and increases soil enzymatic activities [67]. The DOC is a soluble portion of OC which is easily assimilated by microbes in soil, and it was highly and significantly correlated (r = 0.98) with APA and least correlated with urease. The MBC showed the highest value of correlation coefficient (r = 0.97) with DHA and lowest values with urease (r = 0.88). The MBC is considered as one of the principal indicators of the sustainability of the management systems [68]. The MBN exhibited the highest value of correlation coefficient (r = 0.96) with urease activity as compared to rest of the soil properties. The MBC and MBN also were positively and significantly correlated. The DHA showed the highest value of correlation (r = 0.92) with APA among the different enzymes. The above strong correlations indicated a dynamic relationship between the SOC pools and microbial properties. Generally, accrual in SOC pools showed a positive and favourable influence on soil properties. Therefore, nutrient management practices that enhance SOC pools could be beneficial for soil microbial health.

**Table 4:** Correlation between soil carbon and microbiological properties.

	SOC	DOC	MBC	MBN	DHA	Urease	APA	ASA
SOC	1							
DOC	0.937**	1						
MBC	0.975**	0.970**	1					
MBN	0.852**	0.943**	0.922**	1				
DHA	0.952**	0.970**	0.973**	0.935**	1			
Urease	0.777*	0.938**	0.878**	0.957**	0.880**	1		
APA	0.861**	0.980**	0.915**	0.940**	0.922**	0.966**	1	
ASA	0.832*	0.959**	0.883**	0.888**	0.919**	0.934**	0.977**	1

Abbreviations: SOC: Soil organic carbon, DOC: Dissolved organic carbon, MBC: Microbial biomass carbon, MBN: Microbial biomass nitrogen, DHA: Dehydrogenase activity, APA: Alkaline phosphatase activity, ASA: Aryl sulfatase activity.

** Correlation is significant at P < 0.01 level.

*Correlation is significant at P < 0.05 level.

## 4 Conclusions

Long-term application of organic manures and fertilizers in pearlmillet-wheat cropping system for consecutive 25 years increased SOC, DOC, MBC contents, and enzymatic activity. The best combination was the addition of organic manure (FYM) along with NP fertilizers followed by press mud plus NP fertilizers in semi-arid conditions of Hisar, India. Combined use of organic manures: FYM > pressmud > poultry manure in combination with chemical fertilizers was more beneficial for accrual in soil fertility and microbiological properties on a long-term basis than their sole application. However, for MBN and urease activity pressmud was superior to other organics. This differential effect could be due to the variation in SOM added through manures, their nutrient constitution, and the rate of decomposition in soil. A strong relationship between MBC, MBN, and SOC content of soil was observed, indicating a dynamic relationship among these properties. Thus, nutrient management practices that enhance SOC pools could be beneficial for soil microbiological properties. There were lower values of C fractions and microbiological properties in fertilizers treated plots, indicating the deterioration of soil properties over a long period of time. Therefore, the conjuctive use of organics with chemical fertilizers could be the best approach for maintenance of soil health on a long-term basis in pearl millet-wheat cropping systems in semi-arid ecosystems of north-western India.

## Additional information

No additional information is available for this paper.

## Data availability statement

Data will be made available on request.

## CRediT authorship contribution statement

**Manju Kumari:** Writing – original draft, Investigation, Data curation. **Sunita Sheoran:** Writing – original draft, Supervision, Methodology, Conceptualization. **Dhram Prakash:** Writing – review & editing, Visualization, Methodology, Data curation. **Dharam Bir Yadav:** Writing – review & editing. **Parmod Kumar Yadav:** Writing – review & editing, Resources. **Mukesh Kumar Jat:** Writing – review & editing. **Ankit:** Software, Data curation. **Apurva:** Software, Data curation.

## Declaration of competing interest

The authors declare that they have no known competing financial interests or personal relationships that could have appeared to influence the work reported in this paper.
